# Navigation and dance communication in honeybees: a cognitive perspective

**DOI:** 10.1007/s00359-023-01619-9

**Published:** 2023-02-17

**Authors:** Randolf Menzel

**Affiliations:** Fachbereich Biologie, Chemie, Pharmazie, Institut Für Biologie, Freie Universität Berlin, Königin Luisestr. 1-3, 14195 Berlin, Germany

**Keywords:** Cognitive map, Geographic view, Flying insect, Exploratory learning, Generalization, Intuitive model, Radar tracking

## Abstract

Flying insects like the honeybee experience the world as a metric layout embedded in a compass, the time-compensated sun compass. The focus of the review lies on the properties of the landscape memory as accessible by data from radar tracking and analyses of waggle dance following. The memory formed during exploration and foraging is thought to be composed of multiple elements, the aerial pictures that associate the multitude of sensory inputs with compass directions. Arguments are presented that support retrieval and use of landscape memory not only during navigation but also during waggle dance communication. I argue that bees expect landscape features that they have learned and that are retrieved during dance communication. An intuitive model of the bee’s navigation memory is presented that assumes the picture memories form a network of geographically defined locations, nodes. The intrinsic components of the nodes, particularly their generalization process leads to binding structures, the edges. In my view, the cognitive faculties of landscape memory uncovered by these experiments are best captured by the term cognitive map.

## The world views of walking and flying insects

Walking animals are close to the ground and extract predominantly guiding cues above and around them but less or not at all below them. Localized ground-based cues such as odors, elevation and roughness are likely to be used as guides over rather short distances. Besides compasses originating from celestial and earth magnetic cues visual cues originate from the skyline, the panorama and the canopy. The combination of these cues leads to two egocentric learning mechanisms, path integration and image matching (Zeil this volume). The resulting memories and their associations are powerful tools that can be well studied in isolation, e.g., in homing behavior of ants. The dominant experimental approaches have led to the conclusion that ants apply isolated routines of navigation from a “tool box” of behavioral routines of homing (Wehner 2020).

**Fig. 1:**
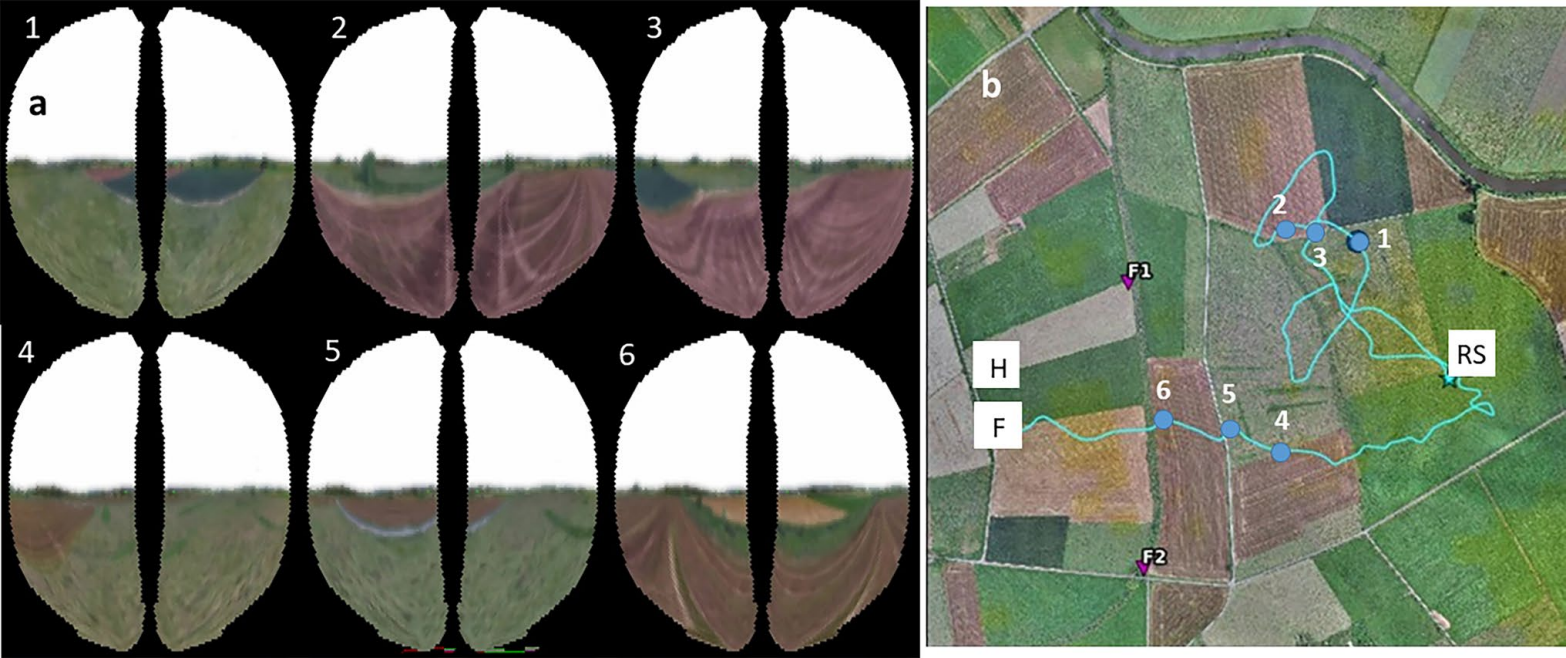
Six animated views of the two compound eyes of a bee flying at 6 m height across an area characterized by grassland, agricultural fields and a structured horizon (50° 48′ 52.21" N, 8° 52′ 20.43" E). The trajectory below shows the points for which these views were simulated for an experimentally measured flight trajectory of a displaced bee. *RS* release site after displacement (start of flight), *H* hive, *F* last radar fix. Courtesy Dr. Landgraf, (Polster et al. 2018). (youtube: https://www.youtube.com/watch?v=sKZZu3AXGfU)

The experience of the landscape differs drastically and fundamentally between walking and flying animals (Fig. 1). The aerial view provides a metric layout of the landscape for each snapshot who’s optical and visual distortions are likely to be resolved in sequential and overlapping images. Walking insects lack the experience of such metric display and need to compose landscape structures from sequences of partial and separate views. Angular modulations of the panorama will play an important role in the world view of walking insects because objects are usually large in comparison to the animal and close. This landscape feature will be of little or no relevance for flying insects given the distance of the objects, they being mostly below the flying insect and the insects’ reduced spatial visual resolution. Particularly salient landscape features become accessible to a flying insect that does not exist for walking insects. Rising landmarks and landmarks on the ground such as trees, bushes, houses, boarders between agricultural fields, rivers, forest edges, and roads. are recognized, uniquely identified, discriminated and learned, can be associated with compass directions, and–as I will argue–become pictures in a cognitive interpretation because the respective images are related to discriminated and learned pictures. Most importantly, such pictures and compounds of pictures bind together in sequential snapshots and their associated compass bearings. Thus memories of aerial views are metric and geographic because they are bound to distant views and one or more compasses. An important component of picture memories is extended (stretched) landmarks. The capacity of recognizing landmarks extending over hundreds of meters or even kilometers requires flight over some distances as in mid-scale navigation, and does not exist in short-range navigation. And it does not exist in walking insects as such. Generalizations about navigation strategies between walking and flying insects have, therefore, to be taken with care and restricted to conditions in the proximity of a goal.

Recognition of pictures of this kind and their associated properties is not just one of the many natural features available to a navigating flying animal but rather a decisive component for cognitive mapping (Jacobs and Menzel 2014). During the construction process of our harmonic radar system we needed to get rough estimates about the flight height of bees. We confirmed the qualitative observations collected during the vanishing bearing experiments that the flight height depended on the distance the bee will fly, the wind conditions and the motivation (whether the bee started a search flight or a straight goal-directed flight). Bees flew below 5 m during flights between the hive and feeder of less than 100 m (hive entrance and feeder about 1 m above ground). Flights further than 300 m led to heights of > 5 m and were still rising within the view of 30 m. Bees could be observed to reach flight height > 15 m within 30 m distance for flights over distances > 1500 m. Queens and drones reached heights > 15 m already during their first 30 m. Thus we made sure that our harmonic radar scanned the bees within our range of 70 cm above the ground to a height of up to 9 m. We estimated that in most of our radar tracking experiments bees flew between 3 and 7 m high.

It has been argued that strategies used to identify and use landmarks close to a goal (the nest entrance, a feeding place) can be used as models to understand navigation strategies in the mid and far range when the flying insect traverses several to many hundreds of meters (Collett et al. 2002, 2006). Although these studies resulted in highly valuable data, they do not capture the essence of the world view in mid and long-distance flights.

My main emphasis is to demonstrate that data exist in navigation studies with flying insects like the honeybee that require the assumption of a topographical representation of meaningful pictures in the natural navigational space, and that “basic models” as presented in e.g., Hoinville and Wehner (2018) and Webb (2019) are not sufficient in particular because bees expect the experience of picture views in the explored area and use these expectations in waggle dance communication.

## The catch and release experiment

**Fig. 2:**
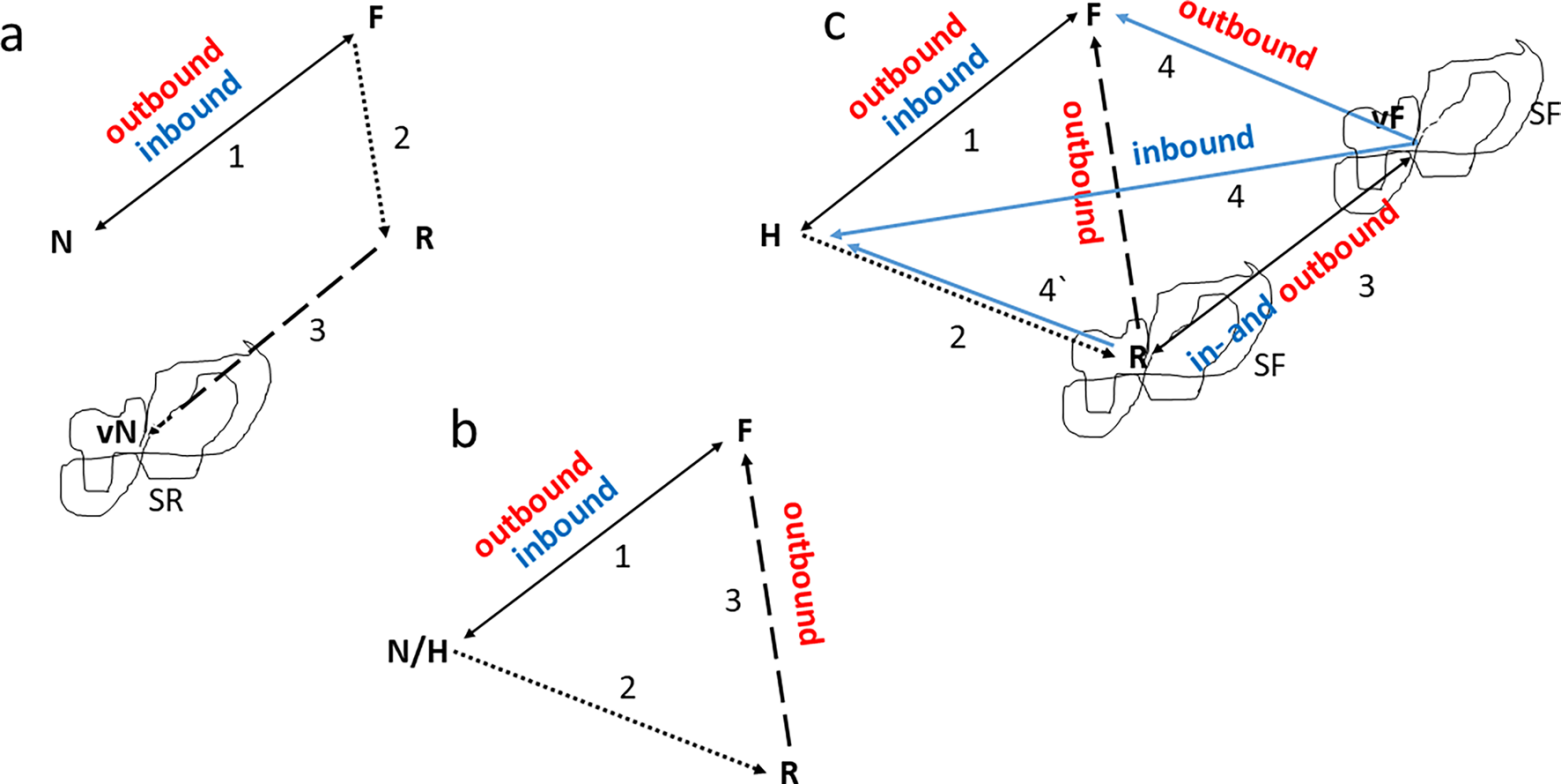
The catch and release experiment. Two designs are shown for the ant Cataglyphis (**a**, **b** (Wehner. 1992, Collett et al. 1999) and one for the bee (**c**, Menzel et al. 2005). **a** The ant was trained to the feeder *F* and then transported from *F* to the release site *R*. She ran the homing vector toward the virtual nest vN, and then started searching (SR) but did not find the nest (*N*). **b** In this experiment the ant was also trained to *F*. When motivated to run to the feeder she was forced to run in a tunnel deviating from the direction toward *F*. When reaching the end of the tunnel at R she ran straight to *F* without searching. Notice that the ant did not perform search runs in this condition. **c** The bee was trained to *F*. When motivated to fly to *F* she was transported to *R* where she flew the vector to the virtual feeder (vF). Notice that the bee made search flights (SF) both at R and vF. Then she chose to perform a straight-homing flight. These homing flights could be initiated at various locations in different bees. No search flights were performed at the feeder. Notice that the bee performed homing flights in both inbound and outbound motivations. Schematized trajectories: 1: trained route to feeder, 2: forced path (ants) or transport to release site (bees), 3: first run/flight after release, 4: homing flights (bees). The trajectory marked with 4 was not observed in the experiment by Menzel et al. (2005) but in other triangulation experiments (see below). Inbound and outbound mark the motivations of the test animals. *N/H* nest or hive, *R* release site, *F* feeder, *vF* virtual feeder, *SR* search run, *SF* search flights

The difference between a walking and a flying insect in the nutshell of navigation can be demonstrated by comparing homing behavior of ants and bees in a paradigmatic test setting, the catch and release experiment. The animals were trained from the nest to a feeder (Fig. 2). Two test conditions are shown for the ant *Cataglyphis* (Fig. 2a, b) and one for the bee (Fig. 2c). In the first test (Fig. 2a, Wehner 1992) the ant was moved from the feeder when motivated to return to the nest. She performed a vector run that would have brought her back to the nest. Then she started to search runs but did not find the nest. In the second test (Fig. 2b, Collett et al. 1999) the ant was forced to run in a tunnel from the nest to a different location from where she moved towards the feeder in straight runs. She did not perform search runs. Other ant experiments are described in Zeil (this volume) that can be explained by image matching and sequential processes as proposed by Cartwright and Collett (1987). In the corresponding experiment with bees (Fig. 2c, Menzel 2005) the animal was also trained to be a feeder. When she was motivated to fly there she was transported in a black box to a release site within her explored the area. She first flew according to the active vector memory (vector from hive to feeder), and then started searching. After terminating her search she applied two strategies. About two third of the tested bees flew straight back to the hive, one third flew first to the feeder and then back to the hive along the trained route. Interestingly only 10% of the bees taking the route via the feeder landed at the feeder and none performed search flights at the feeder, thus nearly all these bees passed the feeder in straight flight (Menzel et al. 2005). In variants of the experimental design reported below it will be shown that bees may also decide to return home from the release site or perform multiple flights between the release site, virtual feeder and real feeder. Thus, the bee applied two or more novel short-cutting flight strategies. Notice that the bee never experienced a vector flight from or to the feeder and the release site or the virtual feeder. The multitude of shortcutting flights as seen in bees (and described further below) has not been found in ants.

**Fig. 3:**
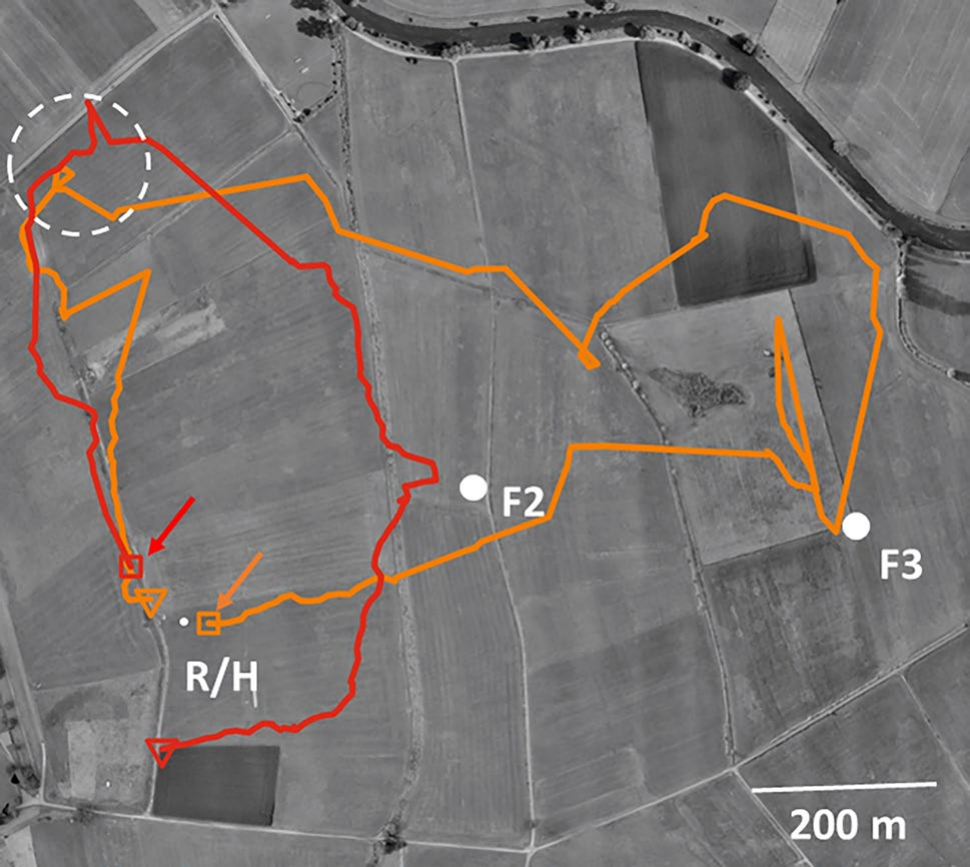
Two examples of flight trajectories of bees that had followed a dance for F2 (red line) or F3 (orange line) and were likely of having foraged before at a natural patch of flowers (broken white line). The arrows mark the starting point of the respective trajectory. The F3 recruited bee flew first towards F3, did not land and then cruised over to the flower patch before returning to the hive (R/H). The F2 recruited bee flew first to the flower patch and then to F2 before returning to the hive

The triangulation strategy in bees appears to be a natural behavior. The two flights shown in Fig. 3 come from bees that had followed waggle dances for feeder F2 or feeder F3. The experiments were performed in an agricultural area with extremely rare natural food sources. A temporary spot of flowers was found in the north of the hive, and marked bees from the observation hive were observed there. One bee flew first to the dance-indicated location and then via the flower spot back to the hive, the other bee flew via the flower spot to the dance-indicated location.

It has been argued (Webb 2019) that ants also perform a shortcut to the feeder. However, the behavior of the ant indicates a vector run that corrected the forced outbound vector, an explanation that does not apply to the bee. True triangulation involving two or several straight runs with changing motivation to my knowledge not been demonstrated for ants. It is essential to consider the motivational level of the animal in the triangulation test. Initially, the animal is in an outbound motivation (move toward the feeder). The ant keeps this motivation and just corrects the forced misdirection. The bee keeps motivation and the related vectorial information, flies to the virtual feeder location (vF), and then starts search flights (Fig. 2c). Most importantly she may or may not switch motivation at vF and fly either back home (inbound), to the feeder (outbound) or to the release site (in- or outbound?) where she again chooses between three potential destinations (see also below).

Triangulation as described here for the bee requires some knowledge about the bee’s location relative to the two other sites, nest, feeder and virtual feeder that can potentially be extracted from large-scale vector integration (Cruse and Wehner 2011) but only if combined with goal specific changes of motivation. The debate about the cognitive map in bees centered on the question of which of the two basic egocentric mechanisms is so well documented in ants or if any of the two apply to the homing behavior of bees via two (or more) paths back to the hive. If she would apply image matching (e.g., of panorama and/or skyline) for both hive and feeder location she could reach the goal by a miss-match reducing mechanism as suggested by Cartwright and Collett (1987). Two observations argue against this hypothesis: (1) the experiments reported by Menzel et al. (2005) were performed in a landscape with a skyline with less than 2° modulation of visual angle and no beacons existed close to the respective locations, (2) the flights were straight and fast. Thus image matching can be excluded (Cheeseman et al. 2014a). If bees would apply large-scale vector integration they need to remember the home-directed vector when starting their shortcuts meaning that local cues at these locations must have been learned during their orientation flights and this location-specific memory needs to be recruited to activate the homing vector. On a formal basis, bees could then integrate this location-specific homing vector with the vector learned during training flights to the feeder and then steer toward the feeder. The question is, how was the home vector associated with the local landscape features, how many such associations exist and are they not only centered at the hive. These are the questions I want to address from the perspective of a flying bee keeping the notion in mind that bees may store picture memories organized in a geographical manner.

## Variants of triangulation in honeybee navigation experiments

Honeybees explore the environment before they start foraging. Initial exploration consists of two components, learning about the immediate surrounding of the hive entrance during a stereotypical scanning behavior (turn-round-and-look-back, Opfinger 1931; Lehrer 1993), and learning during flights into the further landscape (orientation flights, Capaldi et al. 2000; Degen et al. 2015). Multiple orientation flights covering increasing distances and directions are performed, but even a single orientation flight leads to directed homing from the sector explored (Degen et al. 2016). The learning effect becomes also accessible by the finding, that re-orientation flights, flights of bees in a new environment after moving their hive, differ and are less frequent than initial orientation flights (Degen et al. 2018). Furthermore, release within an unexplored sector leads to long search flights during which the bee may cross into the explored sector (and returns) or it may be lost (Degen et al. 2016). As pointed out above, landscape features recognized during entrance scanning and orientation flights differ drastically, and the first does not lend itself as model of the latter. Since bees follow dances only after they had performed orientation flights, it is likely that they calibrate their visual odometer, and learn about the solar azimuth-time function mostly or exclusively during orientation flights (but the evidence is lacking so far).

### Feeder depart: multiple locations and routes of homing flights

**Fig. 4:**
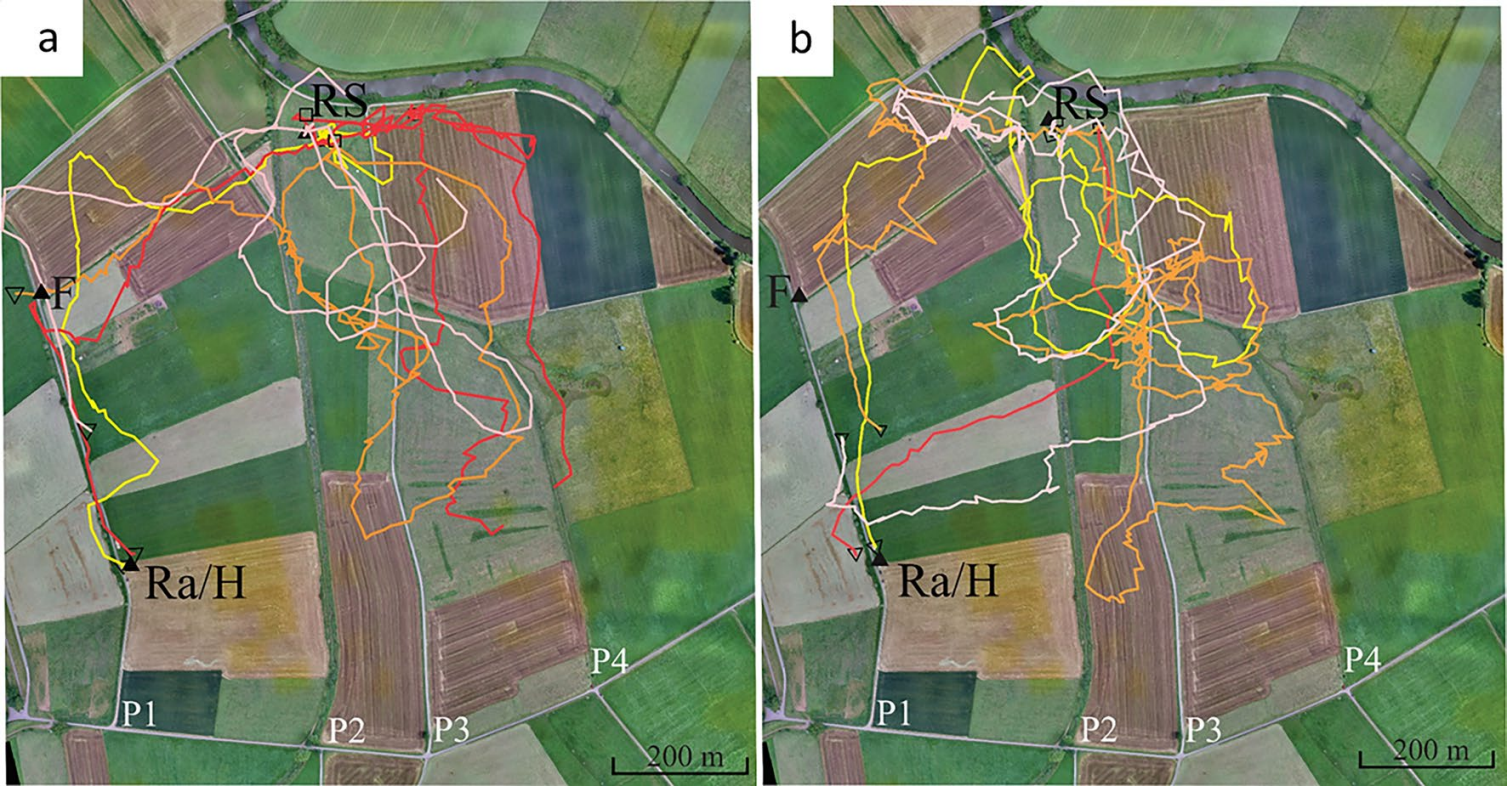
Multiple homing points and routes. The bees were trained along a gravel road (P1) to feeder *F* from the hive close to the radar cabin (Ra/*H*). Feeder departing bees were caught when preparing for homing and released at RS, the release site. The eight representative examples of flight trajectories (different colors for different bees) illustrate the different routes and locations for homing. Notice that the tendency to follow paths (P2, P3) that run parallel to P1. **a** Three of the four bees flew first according to the vector they would have taken at *F*, returned to RS and then via *F* to the hive. One bee (yellow trajectory) flew directly from RS to *F* and then to the hive. **b** Trajectories of four bee, two behaved rather similar to the three in **a** (yellow and orange), two repeated the vector flight and turned toward the hive from the vector flight (red and white). Notice the narrow trajectories to P2 and P3, and the less straight flights when following the “wrong” (unexpected) paths. (from Menzel et al. 2018, Fig. 5)

Figure 4 shows 8 representative flight trajectories of feeder departing bees that were trained to fly along a gravel road (path P1) and were transported to a release site east of the trained route when motivated to return to the hive (Menzel et al. 2018). The experiment was performed in an agricultural area rich of ground structures and panorama. The landscape was chosen because of the parallel running paths (P2–P4). Seven of the eight bees performed first a vector flight according to the learned route, one flew straight back to the hive via the feeder (Fig. 4a, yellow). Two of the bees shown in Fig. 4b (red and white) started their homing flight during their vector flight without returning to the release site. Notice that the flight trajectories were winding when in a search phase and straight when homing. These and multiple examples reported in Cheeseman et al. (2014b), Fischer et al. (2014), Sol Balbuena et al. (2015), Menzel et al. (2005, 2018), Tison et al. (2016, 2017, 2020) showed that feeder departing forager bees started their homing flights at multiple locations during the vector or search part indicating that they switched to the straight homing flight at different locations in the explored area and in different stages of their motivation for homing. These findings support the view that homing flights of well-experienced foragers are not triggered by any particular fixed landscape feature. Rather the bee appears to use vector information and the learned views of landscape features in a surprising variability.

### Feeder depart: shifting the sun compass

**Fig. 5:**
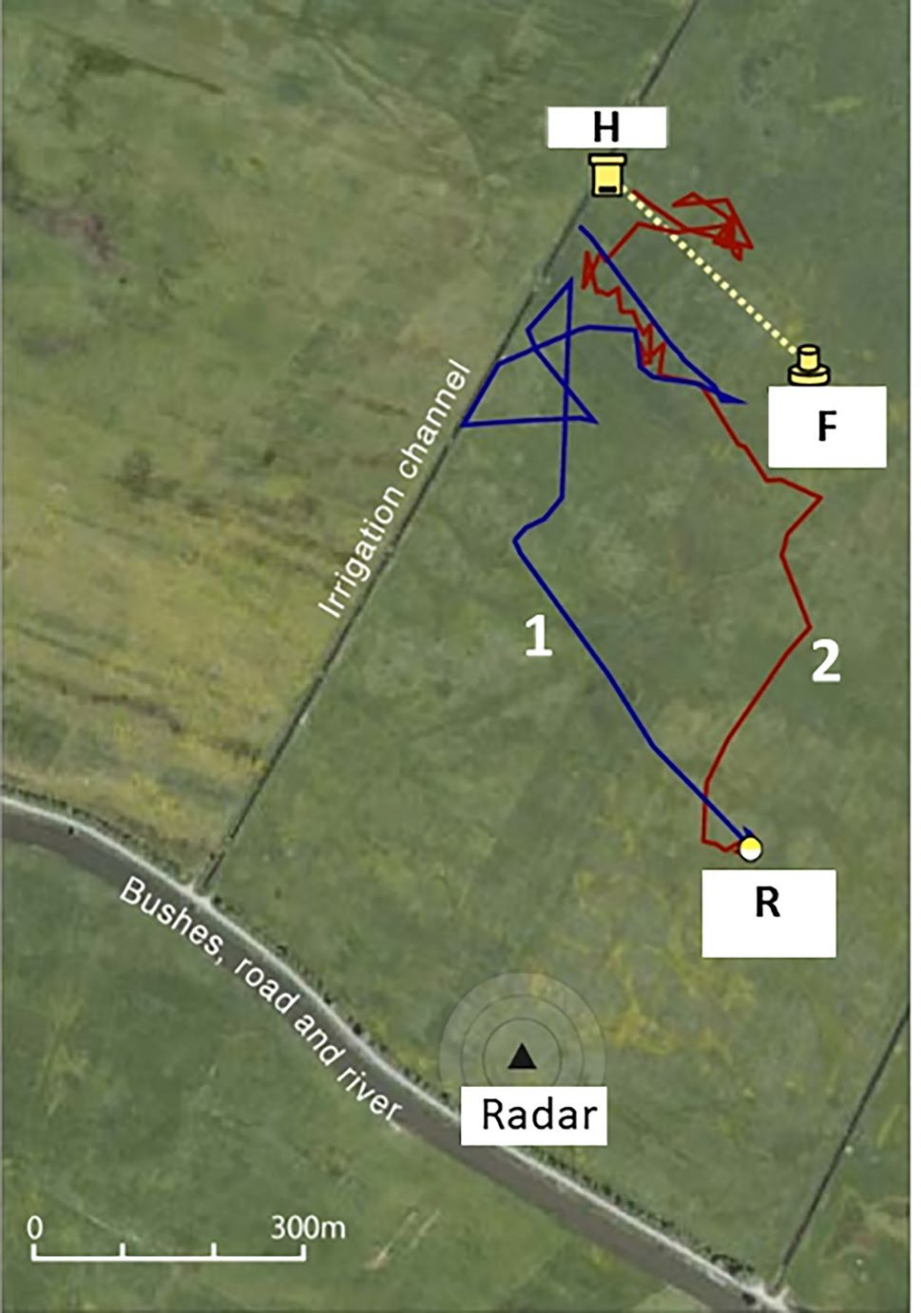
Shifting the sun compass by 6 h of anesthesia. The bees were trained from the hive (*H*) to the feeder (*F*). Two groups of bees were tested. The control group was collected when motivated to fly back to the hive, stored for 30 min in a dark box and then released at *R*. The blue trace marked with 1 shows a representative example of a flight trajectory consisting of the vector flight component and the homing flight component. The experimental bees were collected in the same way, anaesthetized for 6 h and then released at *R*. The red track marked with 2 shows a representative example. The vector flight was shifted to the east, but the bees returned equally well and fast to the hive (from Cheeseman et al. 2012, Fig. 2a)

If foraging bees would have just learned to associate the homing vector with particular landscape features they should be directed to an incorrect location if the sun compass is shifted. We accomplished such a shift by anaesthetizing feeder-trained bees for 6 h with the general anesthetic Isoflurane (Cheeseman et al. 2012, 2014b), and showed that anesthesia slows downs or even blocks the internal clock. Re-synchronization takes hours or even days. Correspondingly the vector phase of the treated foragers was shifted to the east, but homing behavior was not impaired. These experiments were performed in an area that lacked modulations of the skyline and panorama for visual angles of > 2°, and ground structures lacked rising beacons. As in the experiments reported above the switch from the vector to the search components happened at many different locations. Our conclusion was that the initially incorrect flight in relation to the landscape was corrected when the bees switched from the vector flight component to the search and homing components indicating guidance by ground structures that were spatially related to the location of the hive (Fig. 5). If they would have related their homing flights to the shifted sun compass they would have ended up somewhere in the east. If they would have ignored the shifted sun compass their homing flights should not differ from that of the control group. This was found. Although it is known that bees are able to derive sun compass-related flights from the learned landscape if the celestial cues are not available (von Frisch and Lindauer 1954) they relied here on the sun (and blue sky) but only during their initial vector flight. During their home flights, however, they relied on learned ground-based landmarks and ignored the misleading sun compass-related vector. Thus it was concluded that these results together with those cited above in connection to Fig. 4, imply that bees appear to construct an integrated, metric cognitive map. Such a map is metric because it represents directions and distances. It is integrated because it represents diverse landmarks, feeding sources, and terrain features within a single data structure, thereby permits the computation of a course from any represented feature to any other represented feature. This interpretation has been challenged on the basis of two assumptions, the bees might navigate according to a horizontal profile below their visual resolution, and that they may switch back to the actual daytime within a few minutes (Cheung et al. 2014). These arguments were in my view fully rebutted by Cheeseman et al. (2014c) and the basis that no evidence exists for spatial super resolution in bees, and that the readjustment of the circadian rhythm has been shown to last for days.

### Hive depart: trained and dance followed

Honeybees provide the unique opportunity to test two vector memories learned by different means and different motivations, during training at a feeding site and by waggle dancing following. In the latter experiment, a group of individually marked bees was first trained at the feeding site FT, then the site was closed for 2–3 days. During this time the bees explored several times the empty site. On the next day, two bees danced for a different site, the dance site FD, and experimental bees followed these dances. A radar transponder was attached when they left the hive. The locations of the two sites were switched between different groups of bees involved. Four experimental series were run, both sites at distances of either 650 or 300 m either separated by 30° or 60° as seen from the hive (Menzel et al. 2011, Fig. 6). Bees flew from FD to FT or from FT to FD when the distance was smaller than the homing flight (30° separation). When the distance between the two sites was equal to the homing distance (60° separation) they returned to the hive for the 650 m distance but flew to the other site for the 300 m distance. Interestingly, bees flew first to the dance-indicated location FD if they had followed more than 15 dance rounds and to the trained site FT if they had followed less dance rounds. However, even under such conditions they frequently flew to FD in the 30°/300 or 650 m and the 60°/300 m conditions after they had arrived at FT. These experiments were also performed in a landscape without rising beacons and a flat horizon. The feeder for the dancers was hidden in the grass, and in most cases both dancers were in the hive when an experimental bee left the hive.

**Fig. 6:**
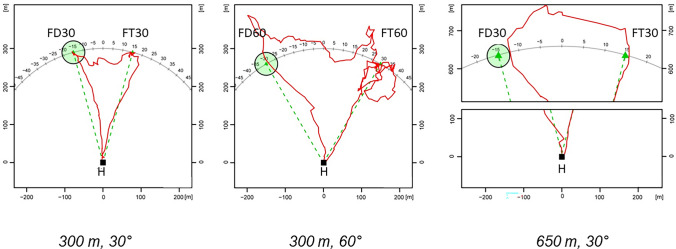
Three representative examples of trajectories of bees that were trained to location FT and later followed dances for location FD after their feeding site FT was closed (Menzel et al. 2011). FD and FT were either at a distance of 300 or 650 m. They also were set either at 30° or at 60° as seen from the hive. The feeder for the two dancing bees (FD) is marked with a green circle. The direct connections between the hive *H* and FD or FT are marked with a green dotted line. The shortcut between FD and FT is not marked. The flight over 650 m is broken in two subfigures (right figure)

Taking these data on their own one may again argue that the bees performed large-scale path integration as suggested by Cruse and Wehner (2011). However, such a form of path integration differs substantially from dead reckoning since memories have to be fed into a mechanism that performs a triangulation process based on information gathered under completely different conditions, flight experience and waggle dance following. The memory structure underlying such decisions needs to include the spatial references of the three sites (hive, FD and FT) including their respective distances. Furthermore, bees had to make decisions between returning to the hive or flying the shortcut, a decision that involves different motivations (outbound vs inbound).

### Hive depart: dance followed and translocated

**Fig. 7:**
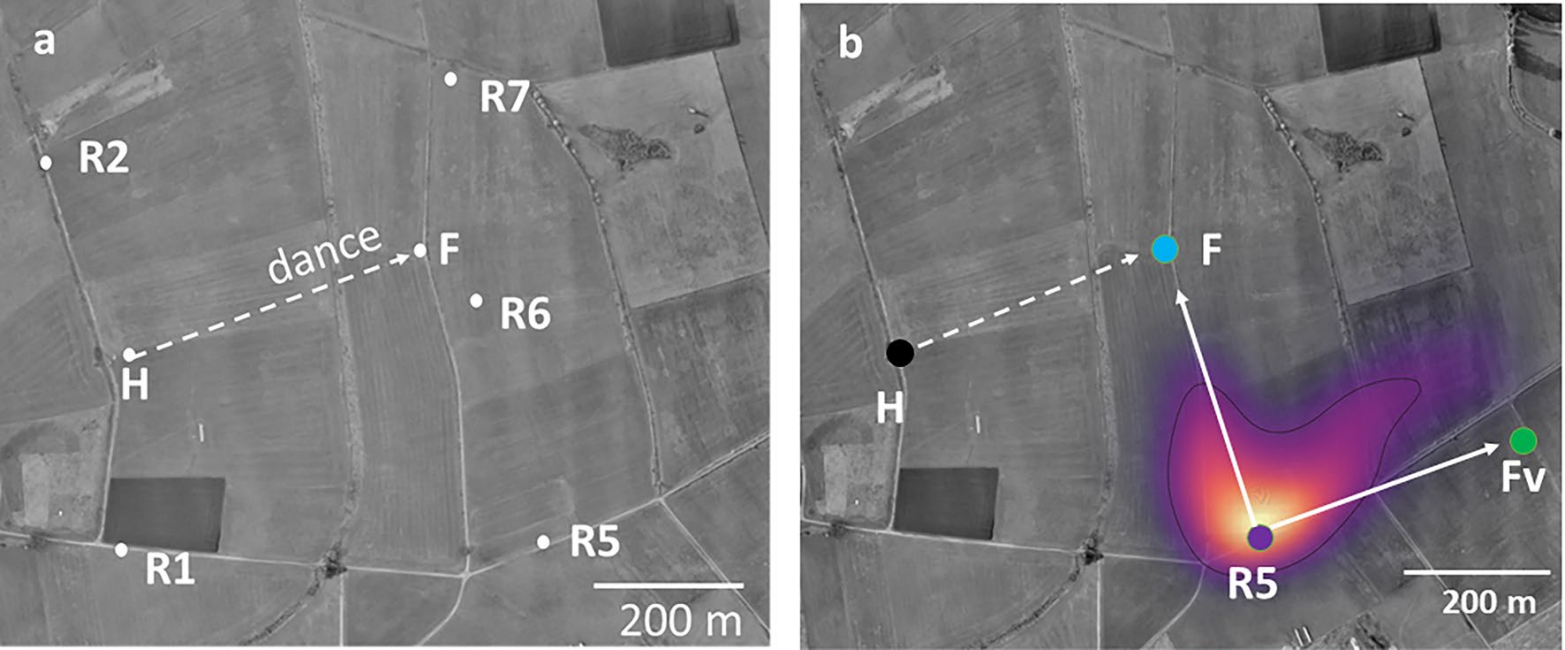
Dance followers search for the dance-indicated location not only via the vector information transmitted in the dance but also toward the real location independent of where they started their search flights (Wang et al. 2022). **a** experimental design. *H* hive where the dancer performed the dance for feeder *F* (dance information: broken line). **b** heat map of the search flight fixes of dance flowerers that were release at R5. *F* feeder for the dancer, *Fv* the virtual feeder for the dance follower if she would follow only the flight instruction of the dance

In a recent experiment, we addressed the question of whether bees that had followed a dance are guided only by the flight instruction (the vector information communicated by the waggle dance) or toward the dance-indicated location irrespective of where they started their flight (Fig. 7, Wang et al. 2022). The experiments were carried out in an agricultural landscape, and the distance between the hive and the dance indicated location *F* was 388 m. The experimental design included 5 release sites other than the hive, three of them further away from the hive than the feeder for the dancers. The dance followers performed drastically different vector flights at the different release sites and then searched toward the real feeder location of dancer *F*. Figure 7 shows the result for one of the 5 release sites, R5, expressed as a heat map of all search flight fixes. All flights and analyses can be found in Wang et al. (2022). We concluded from the analyses of several hundred flights that dance-following bees use two sources of information to guide their flights, the vector information communicated by the dancer and the information derived from the endpoint of the vector embedded in their navigational memory.

## Memory processing and generalization of landscape memory

Memory results from learning but it does not exist in its final form immediately after learning. Rather memory is processed, shaped and changed over time and in the context of further experience. The dynamics of the memory in honeybees following reward learning have been documented under multiple conditions (Menzel 2022). Learning of landscape memory is most likely based on observational (exploratory) learning (Degen et al. 2016). A strong hint for memory processing of landscape memory comes from the observation that consolidation of new landscape memory including waggle dance communication requires undisturbed sleep (Klein and Seeley 2011; Beyaert et al. 2012).

The ability to generalize (rather than to discriminate) is a key component of cognition bound to phenomena like selective attention, expectation, categorization, similarity judgments and saving (Rescorla 1992; Blaisdell 2008; Zentall et al. 2008). “Generalization occurs in learning, and is essential for deriving knowledge from experiences and for skills of all kinds. It is the basis of predicting future situations from past experience and for drawing analogies.” (Gregory and Zangwill 1987, p. 284). Thus generalization speaks to the cognitive dimensions of using memory for solving a problem. In the case of landscape memory, pictures learned at a particular site (as introduced above) will be transferred to another site depending on the similarities of these learned pictures. Thus partly overlapping memory contents of spatially closely related sites will lead to some measure of neighborhood connecting these sites. This property of memory processing is a central component of the model presented below.

Bees gain from generalization when environmental conditions alter the landscape features or when animals move to a new nest site because they use relevant memory and ignore irrelevant memory. In essence, memories are retrieved, compared with the perceived stimuli that are then evaluated according to the needs of the animal. We set up an experiment in which the needs were well defined and the generalization could be quantified (Bullinger et al. inpress). The animals were collected at a feeder when they had sucked their fill and were motivated to return to the nest very close to the feeder, thus the locations of the hive and feeder were practically identical. In our experiments, bees from 5 different home areas were transferred to the same test area that differed more or less from the home area. We expected that the animals would perform random search flights with multiple returns to the release site or stereotypical flights similar in bees from the different home areas if they did not recognize any even partially suitable picture memory. If, however, their navigation memory could be at least partially generalized to the experienced pictures seen in the test area their behavior would differ from random and stereotypical search. We argued that generalization between home area-specific memories and experienced pictures in the test area would motivate them to explore the related landscape features. The density of exploration and specific patterns of their search flights may thus reflect a generalization effect. We found that searching was not random and differed between the animals from the different home areas. Next, we analyzed multiple components of the search flights (directions of flights, density of fixes, guidance by elongated ground structures) and applied a partial least squares regression (PLS) analysis for a similarity-difference gradient of the unknown guidance parameters. We chose PLS over principle component analysis (PCA) because it is the method of choice to find the relationship between two spatial matrices since it captures the covariance structures of two spaces. Classification was performed with a support vector machine to account for optimal hyperplanes. The modeled groups were well separated from the experimental groups indicating that random or stereotypical flights contribute only partially. Home areas were separated according to their differences in the layout of the extended ground structures. These findings clearly indicated a navigational memory that is processed as a unique cognitive component as other memories and should be understood as such.

## A graph theory-based model of honeybee navigation

In the following, I propose basic components of navigational memory in the honeybee based on our experiments described above. The essential and elementary component is a node composed of the metric picture learned at a selected location and associated with intrinsic egocentric parameters. I argue that nodes are connected by edges resulting from generalization processes. This intuitive approach is motivated by studies in robot navigation like the SLAM (simultaneous localization and mapping) procedure (Durrant-Whyte and Bailey 2006) and “topometric maps” (Badino et al. 2012). As in these studies, the sequential appearances of nodes and the way of connecting them via edges leads to the construction of topographical elements that form a map in which the bee localizes herself and “plans” shortcuts not only between experienced locations but also between locations communicated symbolically by the waggle dance. While multiple vector memories associated with experienced locations would be formally sufficient to construct a map (Webb 2019), I argue that the bee uses her power to store and use large amounts of picture memories as documented in visual discrimination, generalization and rule learning experiments (Menzel et al. 2007; Srinivasan 2010; Giurfa 2015, 2019). In my view, the secret of the cognitive map in a flying insect like a bee cannot be uncovered by focusing on ad hoc neural processing like image matching or/and path integration. While ad-hoc processes are most likely performed in the central complex of the central brain (Pfeiffer and Homberg 2014; Seelig and Jayaraman 2015), the complex analyses proposed here are rather based on memory processing going on in neural nets spanning the whole brain with a focus on visual ganglia and the mushroom bodies. The hypotheses presented in the following are intended to structure the data collected as exposed above and to formulate new experimental approaches for the future. In particular, new questions will arise that can be posed to the extrinsic neurons of the mushroom body.

**Fig. 8:**
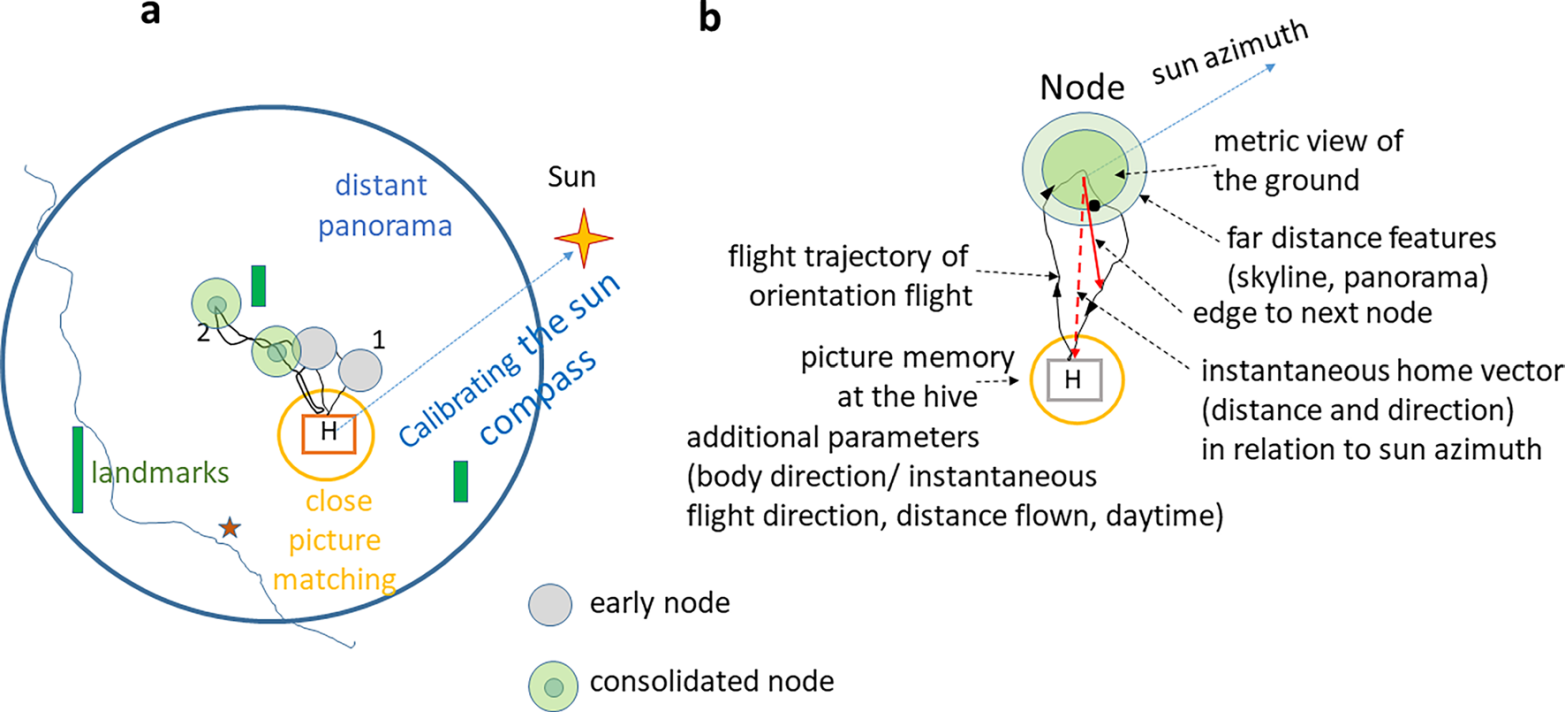
Illustration of the basic conditions a bee finding herself in when leaving the hive for the first times. The distinction between early and consolidated nodes captures the dynamic of memory formation (see text). **a** She first performs short scanning flights in the close vicinity hovering around the hive entrance and facing it multiple times from different directions (yellow circle, “turn-round-look-back” behavior Opfinger 1931; Lehrer 1993). The first orientation flight is usually rather short (trajectory 1). The second orientation flight reaches further distances, and may vary in direction (Capaldi et al. 2000; Degen et al. 2015). **b** The elementary component of exploratory memory is a node consisting of the picture memory with its components (metric view of the ground, additional environmental cues, egocentric parameters such as the instantaneous home vector, the time of the day, the celestial cues and the motivational state)

The behavioral transition from scanning the immediate surrounding of the hive to exploring the further area by a young bee is a sudden turn away from the hive entrance, an acceleration of speed and the beginning of a fast and straight flight (personal observation and radar measurements). Now the bee will be exposed for the first time to an aerial view together with views of the panorama and the solar cues (sun and blue sky with polarized light) (Fig. 8a, b). Egocentric path integration will ensure a safe return to the hive. Angular changes of both landscape views and sky cues are strictly in phase with the metric picture memory as defined above. Elevated objects at different distances will differ in motion parallax, a feature easily used for signaling an early and uncalibrated distance measure, possibly like in conditions of close object distance estimation (Lehrer 1994; Lehrer and Collett 1994). The stereotypical distance measure based on optic flow (Shrinivasan et al. 2000) will be augmented by learning the sequences of landmarks (Menzel et al. 2010).

**Fig. 9:**
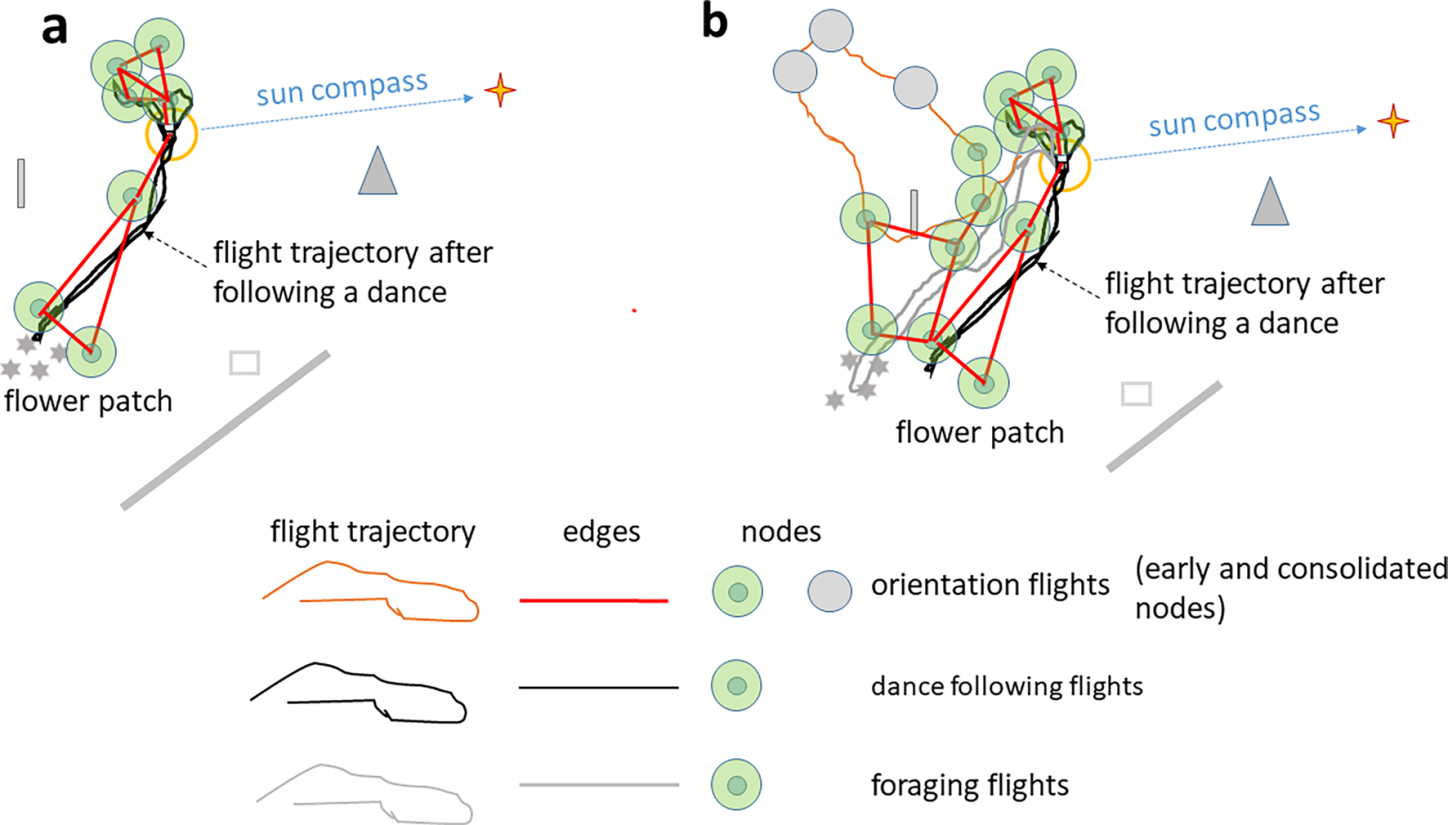
Forming edges between nodes by a generalization process. The distinction between early and consolidated nodes captures the dynamic of memory formation (see text). **a** Waggle dance following leads to a directed search flight guided by the calibrated sun compass and the calibrated visual odometer and the vector information from the dance. **b** Foraging flights after successful arrival at the forage (grey trajectory, edges and nodes) and additional orientation flights (brown trajectory) add nodes and edges). The inset illustrates the three categories of learning about the landscape features leading to nodes that are connected by a generalization process across learning categories. The grey triangle indicates a distant landmark

Multiple views are experienced during the first orientation flight but a salient condition will be necessary to establish a localized picture memory, a node. Such a condition could be a change of motivation, e.g. the decision to return to the hive. A node could also be initiated by a particular conspicuous landmark. The node structures the memory of a location characterized by its intrinsic metric components and the egocentric components associated with it, the current setting of the path integrator (home vector), the sun azimuth, time of the day, additional features of the landscape (e.g., the panorama or a close object). Prominent ground structures, such as elongated paths, are particularly salient and informative features. Furthermore, a node carries the potential to hook up to another node by storing an edge (connecting line) to the next-to-be-established node or to nodes from earlier explorations. The essence of this proposal lies in the assumption that the basic elements of navigation memory are stored aerial picture memories with their intrinsic geographic properties. The dynamic of memory formation including the role of sleep (Beyaert et al. 2012) and the transition from short-term to long-term memory (Menzel 2014) is captured by the distinction between early and consolidated nodes (Figs. 8 and 9).

**Fig. 10:**
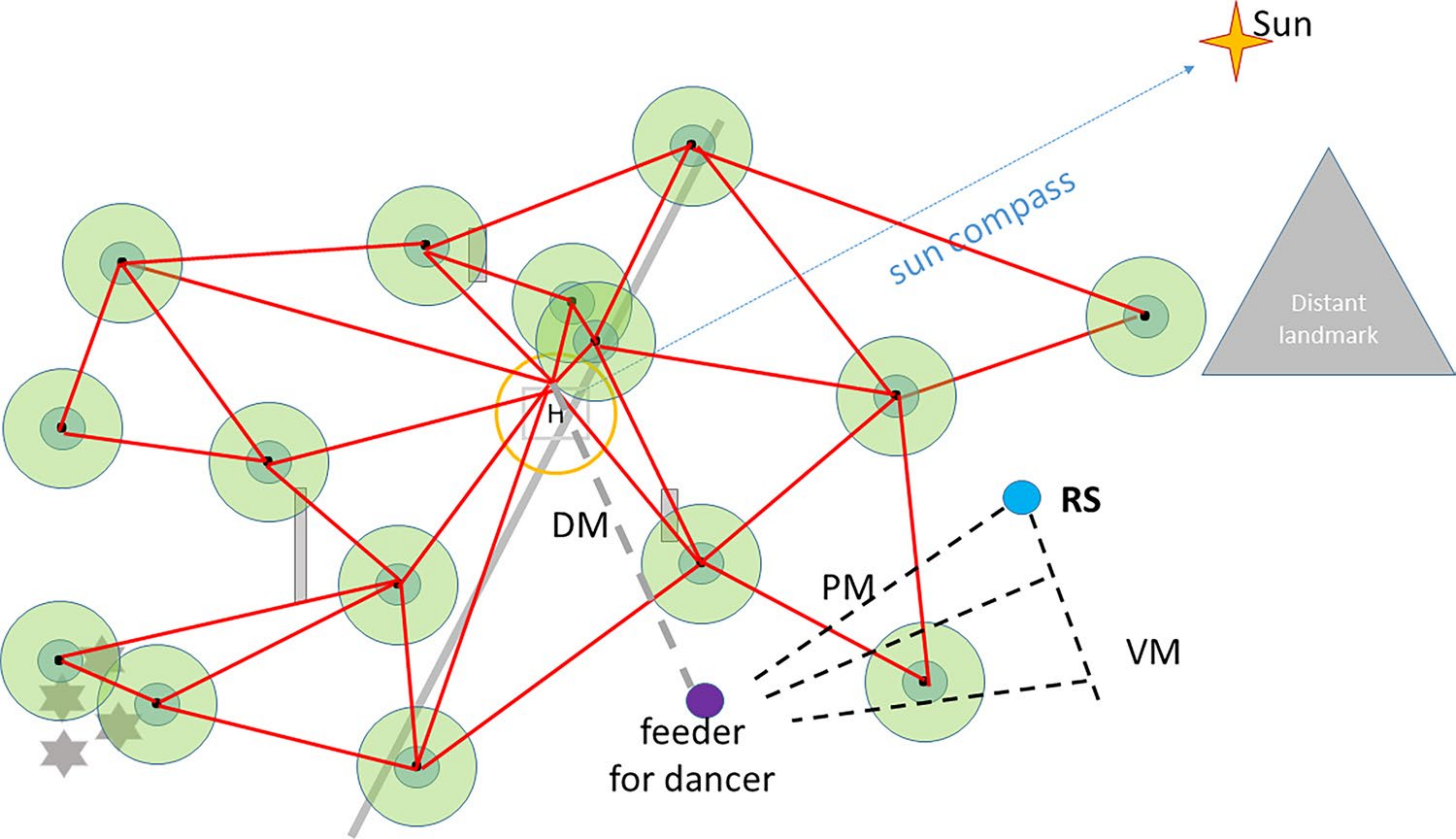
Structure of the landscape memory addressed in waggle dance communication. The dance message (DM) is depicted by the broad broken grey line. Dance followers are released at the release site RS. They apply two search strategies: they partly follow the vector message (VM) by flying a changed vector flight and they fly toward the true location from different places during their search (broken black lines, place message, PM)

Edges are formed by a generalization process. Nodes connected by short edges have partially overlapping properties, and they are connected by generalization. The overlapping properties of their respective memories are depicted in Fig. 10 by partially overlapping nodes. As reported above, the generalization of landscape memory has been demonstrated recently by testing bees from different home areas in a novel environment (Bullinger et al. inpress). In learning theory generalization is the counterpart of discrimination. In discrimination experiments, animals are trained to distinguish between differentially trained stimuli. To test generalization, however, animals are asked whether and how they transfer what they have learned to other stimuli they are able to discriminate. The transfer is either immediate and specific or used later for new learning, e.g., for extracting a hidden category as in rule learning. The latter has been found in bees multiple time in the context of reward learning (e.g., learning of bilateral symmetry, Giurfa et al. 1995), and matching-to-sample (Giurfa et al. 2001). Learning about a stimulus influences strongly how a behavior is generalized to other stimuli (Blough 1975; Kehoe 2009). Generalization appears not only between the visual components of the picture memories but also between the associated contents e.g. homing vector, sun compass, daytime, and serial distance measure. The topographic alignment of the nodes and their edges is an essential component of the calibration process of the sun compass and its dependence on the local ephemeris function. Only stable geographic conditions involving several to many nodes and their edges will allow to derive the solar ephemeris function.

Symbolic transfer of vector information via the waggle dance will be possible after the sun compass and the odometers have been calibrated (Figs. 9a, 10). Both measures require reference to stable geolocations. Successful homing after translocation to already explored areas does not prove a topographic representation of navigational space. However, it has been shown that eliminating reference to the sun compass still allows successful homing (see above). Multiple foraging flights and additional orientation flights were found to be interspersed between foraging flights (Degen et al. 2015) and thus add nodes and edges (Fig. 9b).

A dancing bee transmits vector information to the dance follower (Fig. 10). As shown in Wang et al. (2022), dance followers not only refer to the vector information in their search flights but also to information about the true location of the dance vector endpoint derived from their spatial memory. Search flights directed toward the true location are initiated at various locations during the vector flight or return flights to the release site. This finding has been interpreted to show that dance followers translate the communicated vector into location-specific information. Furthermore, dance followers expect landscape features when decoding the dance message. Evidence comes from the observation that dance followers change their vector flight components (shorter distance and altered direction) if exposed to landscapes that do not match the expected landscape, and they extend their searches along elongated ground structures if the dancer had experienced a similar structure pointing approximately in the same compass direction (unpublished observation). Control experiments excluded the possibility that the dancer had a spontaneous tendency to follow the expected landscape structure (an elongated ground structure). Thus both the location of the dance indicated place as well as the features on the way to it are construed by the dance follower from the vector information of the dance. Since dancers and dance followers are experienced bees that have their landscape memory established and their sun compass as well the odometer calibrated it is legitimate to assume that cognitive capacities found in dance followers will also apply to dancers.

## Conclusion: how much cognition in the bee brain?

I argue that the landscape memory unifies the multiple components of spatial reference to a unique cognitive entity based on rule learning similar to other forms of memory processing in honeybees. The components can be separately studied in well-designed experiments, but these approaches loose the cognitive dimensions of landscape memory. Furthermore, generalization from experiments in the close range at the hive entrance and the feeding site do not capture the unique conditions in the navigation range and thus are not meaningful for conditions in which picture learning as understood here provides the essence of navigation in the mid-range. Cognitive dimensions of landscape memory in flying insects like the honeybee include faculties like the expectation of specific landscape conditions, generalization across deviations from the learned pictures, transfer between different sequentially learned landscapes, expectation about landscape features and decision between two or multiple goals. We have focused our studies on these aspects of landscape memory and find cogent evidence in favor of these cognitive dimensions including the expectation of landscape features that the animal extracts from its landscape memory via waggle dance communication. I find it justified to capture these unique conditions with the term cognitive map.

The search for the neural correlates of the cognitive map has to include brain structures like the mushroom bodies as the sites of high-order convergence and integration (Filla and Menzel 2015; Menzel 2014, 2022). Other parts of the brain like the visual, olfactory and mechano-sensory first and high-order processing parts of the brain together with neural circuits involved in feedback and efference copy will certainly be of upmost importance in understanding the neural processes but the unique properties of the cognitive map memory require in my view the combinatorial power of the mushroom bodies.

## Data Availability

The data that support the findings of this study are available from the corresponding author upon reasonable request.
